# Two Genetically Distinct Lineages in Galician Populations of the Three‐Spined Stickleback (*Gasterosteus aculeatus*)

**DOI:** 10.1002/ece3.74051

**Published:** 2026-07-19

**Authors:** Yo Y. Yamasaki, Yu Endo, Naomi Musto, Juan Galindo, Alberto Velando, Sin‐Yeon Kim, Atsushi J. Nagano, Asano Ishikawa, Jun Kitano

**Affiliations:** ^1^ Ecological Genetics Laboratory National Institute of Genetics Mishima Shizuoka Japan; ^2^ Genetics Course The Graduate University for Advanced Studies Shizuoka Japan; ^3^ Centro de Investigación Mariña Facultad de Biología, Universidade de Vigo Vigo Spain; ^4^ Grupo Ecoloxía Animal, Torre CACTI, Centro de Investigación Mariña Universidade de Vigo Vigo Spain; ^5^ Bioscience and Biotechnology Center Nagoya University Nagoya Aichi Japan; ^6^ Institute for Advanced Biosciences Keio University Tsuruoka Yamagata Japan

**Keywords:** convergent evolution, Iberia, local adaptation, parallel evolution, phylogeography

## Abstract

The three‐spined stickleback ( *Gasterosteus aculeatus* ) is a widely used model for studying the genetic basis of phenotypic variation. However, genomic studies have focused primarily on a limited number of geographic regions, leaving some regions underrepresented. In this study, we investigated genetic variation in stickleback populations from Galicia in northwestern Spain, a region near the southern limit of the species' European distribution that has been proposed as a glacial refugium but remains underrepresented in genomic analyses. Using double‐digest RAD sequencing, we identified two genetically distinct lineages within Galician populations. These lineages showed substantial genetic differentiation (mean *F*_ST_ = 0.435) and also exhibited fine‐scale population structure within each lineage. We detected morphological variation among Galician populations, although its association with lineage identity and ecology remains unclear. Most individuals carried the low‐plated allele at a major armor plate locus (*Eda*), which is commonly found in freshwater populations throughout the species' range. Using whole‐genome sequencing, we showed that both lineages were assigned to the Southern European Clade of the three‐spined stickleback. Our results reveal previously unrecognized phylogeographic structure within Galician sticklebacks and highlight the importance of sampling underrepresented regions in model systems. These populations provide a useful system for investigating how geographic structure shapes patterns of genetic and phenotypic variation.

## Graphical abstract

Two genetically distinct lineages of three‐spined sticklebacks occur in Galicia, Spain. Both belong to the Southern European Clade of the three‐spined stickleback.

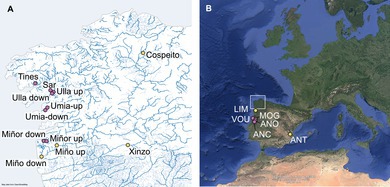

## 1 Introduction

Organisms with broad geographic distributions that occupy a wide range of environments are ideal models for investigating how historical and contemporary processes shape patterns of genetic and phenotypic variation (Booker et al. 2021; Fan et al. 2016; Kawecki and Ebert 2004). For example, patterns of phenotypic and genetic convergence are influenced by underlying population structure and past and present gene flow, which determine how genetic variation is distributed across regions (Kawecki and Ebert 2004; Stern 2013; Richardson et al. 2014). Therefore, populations from geographically distinct regions, particularly those that remain underrepresented in genomic studies, provide important opportunities to investigate how genetic structuring shapes phenotypic and genetic variation.

The three‐spined stickleback ( *Gasterosteus aculeatus* ) is a well‐established model system for studying the genetic basis of phenotypic variation. This species is widely distributed across coastal regions of the Northern Hemisphere and has adapted to a diverse range of aquatic environments (Bell and Foster 1994; McKinnon and Rundle 2002; Schluter and Conte 2009; Wootton 1976). Its extensive phenotypic diversity has made it a powerful system for investigating patterns of phenotypic variation and their genetic basis (Bomblies and Peichel 2022). Comparative studies across geographic populations have shown that adaptation to similar environments often leads to the evolution of similar phenotypes (Bell and Foster 1994; Schluter 2000). Furthermore, the genetic basis of these convergent traits can be shared across populations in some cases, while differing in others (Coll‐Costa et al. 2024; Colosimo et al. 2005; Fang et al. 2020; Ishikawa et al. 2019; Jones, Chan, et al. 2012; Jones, Grabherr, et al. 2012; Yamasaki et al. 2019).

However, most of these studies have focused on populations from a limited set of geographic regions, such as the northeastern Pacific, northern Europe, and the Japanese Archipelago. Although several studies have examined phenotypic variation in southern European populations (Fernández et al. 2000; Hermida et al. 2005; Lucek and Seehausen 2015; Prieto et al. 2005; Rind et al. 2020; San Miguel et al. 2020; Zanella et al. 2015), few genomic studies have addressed this region (Coll‐Costa et al. 2024). Furthermore, populations from the coastal region of Galicia in northwestern Spain remain underrepresented in genomic studies of sticklebacks, despite their importance as rear‐edge populations for understanding evolution under current climate change in glacial refugia (Perrier et al. 2026).

Previous studies have characterized the genetic structure of stickleback populations in the Iberian Peninsula. A phylogenetic study of Spanish populations based on mitochondrial and microsatellite markers showed that several populations from the Miño and Limia Rivers in Galicia are genetically similar to Portuguese populations (Vila et al. 2017). More recent genomic work has explored variation in southern European populations (Coll‐Costa et al. 2024), but did not include coastal populations from Galicia, leaving the phylogeographic structure of this region uncharacterized. In a broader‐scale analysis of southern European populations based on microsatellite markers (Lucek and Seehausen 2015), only a single Galician population from the upper Miño River was included; this population was phylogenetically close to those from Corsica and Sardinia. As a result, both the phylogeographic structure within Galicia and the position of these populations within the broader European lineage structure remain unclear. Furthermore, a global‐scale phylogenomic analysis identified three main clades of the three‐spined stickleback: the Southern European Clade, the Trans‐Atlantic Clade, and the Trans‐Pacific Clade (Fang et al. 2018). However, this analysis did not include any Galician populations.

To address these gaps, we conducted double digest restriction‐site associated DNA sequencing (ddRAD‐seq) of stickleback populations sampled across Galicia, complemented by whole‐genome sequencing (WGS) of a subset of individuals. We then analyzed genetic differentiation among Galician populations and examined their phylogenetic relationships in the context of global stickleback diversity. Our analyses revealed the presence of two genetically distinct lineages within Galicia, both of which belong to the Southern European Clade, providing new insight into fine‐scale phylogeographic structure in this region.

## 2 Materials and Methods

### 2.1 Fish Sampling and DNA Isolation

**FIGURE 1:**
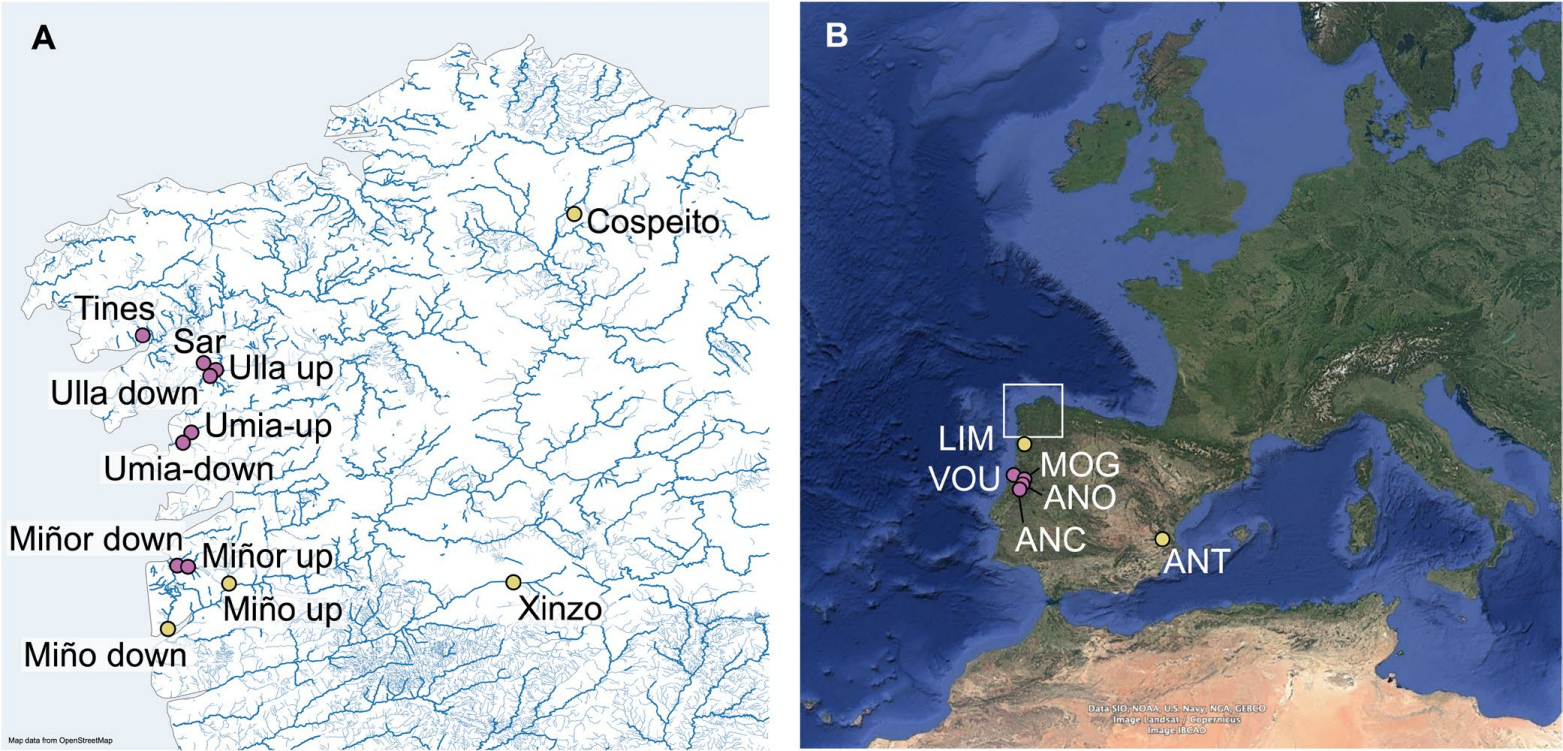
Sampling sites. (A) Sampling locations in the Galicia region. Circle colors indicate two lineages identified in this study (see Figure 2): Group 1 (purple) and Group 2 (yellow). Rivers and streams were mapped using QGIS v3.44 (https://zenodo.org/records/19401570) (Dawson et al. 2026). Hydrographic data were obtained from OpenStreetMap via the Geofabrik download service (https://download.geofabrik.de/) and from Natural Earth (https://www.naturalearthdata.com/), accessed in April 2026. (B) Sampling locations across the Iberian Peninsula from a previous global study (Fang et al. 2018). Circles are colored according to lineage assignment (Group 1 in purple and Group 2 in yellow; see Figure 5). The Galicia region is indicated by a white box. A regional overview map of Spain was created using Google Earth Pro v7.3.7.1155 (https://www.google.com/earth/).

We sampled 228 individuals from 12 locations in the Galicia region of northwestern Spain using hand nets (Table 1; Figure 1). Wild fish were sampled with permission from the Consellería de Medio Ambiente, Territorio e Vivenda of the Xunta de Galicia (EB‐101/2015, 111/2016, and 083/2019). Ten sampling sites were located near the Atlantic coast, whereas two sites were located inland. The migratory life history (i.e., anadromous or freshwater‐resident) of these populations is currently unknown. Salinity measured at a subset of sampling sites in June 2026 approximately 2.5–3 h after high tide ranged from 0 to 6 ppt: 6 ppt at Miño downstream, 2 ppt at Miñor downstream, and 0 ppt at Miñor upstream and Umia downstream. Genomic DNA was extracted from pectoral fin tissue using the DNeasy Blood & Tissue Kit (Qiagen, Hilden, Germany).

**TABLE 1:** Sampling locations of Galician three‐spined sticklebacks used in this study.

Location ID	River basin	Latitude	Longitude	*N*	Date of collection
Tines	Entíns	42.8394	−8.90644	22	2016‐11‐23
Sar	Ulla	42.72126	−8.66663	17	2013‐02‐05
Ulla downstream	Ulla	42.71678	−8.69136	19	2020‐02‐17
Ulla upstream	Ulla	42.71906	−8.67811	4	2020‐02‐17
Umia downstream	Umia	42.50118	−8.77763	22	2016‐11‐23
Umia upstream	Umia	42.53455	−8.75557	21	2016‐11‐12
Miñor downstream	Miñor	42.11172	−8.79406	21	2016‐11‐14
Miñor upstream	Miñor	42.10973	−8.76656	22	2016‐11‐11
Miño downstream	Miño	41.9126	−8.83076	20	2016‐11‐25
Miño upstream	Miño	42.05638	−8.63426	24	2016‐11‐28
Cospeito	Miño	43.27497	−7.60547	10	2013‐02‐05
Xinzo	Limia	42.05907	−7.72767	26	2013‐02‐05

### 2.2 ddRAD‐Seq Data Analysis

ddRAD‐seq libraries were prepared using EcoRI and BglII following previously described protocols (Kakioka et al. 2020; Sakaguchi et al. 2015). Libraries were sequenced on an Illumina HiSeq X platform by Macrogen Japan. Sequence data are available from DDBJ (see Table S1 for the accession numbers). As an outgroup for phylogenetic analysis, we used ddRAD‐seq data from a single individual collected from Lake Washington, North America (accession DRR593529) (Yamasaki et al. 2026).

Raw reads were filtered and trimmed using fastp v0.23.2 (Chen et al. 2018) with the following parameters: ‐‐detect_adapter_for_pe, ‐‐cut_right, ‐‐cut_window_size 4, ‐‐cut_mean_quality 20, and ‐l 35. The filtered reads were mapped to the stickleback version 5 reference genome (Nath et al. 2021) using bwa‐mem2 v2.2.1 (Li 2013; Md et al. 2019) with default settings. Single nucleotide polymorphisms (SNPs) were called using bcftools v1.23 (Danecek et al. 2021) with default settings.

For phylogenetic analysis, low‐quality SNPs were filtered using vcftools v0.1.16 (Danecek et al. 2011) with the following parameters: ‐‐max‐meanDP 87.5, ‐‐minDP 8, ‐‐minQ 20, ‐‐minGQ 20, ‐‐min‐alleles 2, ‐‐max‐alleles 2, ‐‐mac 1, and ‐‐max‐missing 0.8. The threshold for maximum mean depth (87.5) was calculated as mean depth + 4√mean depth (Li 2014), based on sites with depth ≥ 1 and missing rate ≤ 20%. Individuals with a missing rate exceeding 50% were removed using PLINK v1.90b6.21 (Chang et al. 2015), resulting in a final dataset of 223 individuals. Variant files were converted from VCF to PHYLIP format using vcf2phylip.py v2.8 (Ortiz 2019), and invariant sites introduced during filtering were removed using ascbias.py (https://github.com/btmartin721/raxml_ascbias). Phylogenetic analysis of 4965 SNPs was performed using RAxML‐NG v1.2.2 (Kozlov et al. 2019) with GTGTR+G + ASC LEWIS model. Branch support was assessed using 200 bootstrap replicates with the standard Felsenstein bootstrap approach (Felsenstein 1985).

For calculation of pairwise *F*_ST_, we first filtered SNPs using vcftools (v0.1.16) (Danecek et al. 2011) with the following parameters: ‐‐max‐meanDP 87.5 ‐‐max‐alleles 2 ‐‐minQ 20 ‐‐not‐chr chrM ‐‐not‐chr chrY ‐‐not‐chr chrXIX ‐‐not‐chr chrUn ‐‐minDP 8 ‐‐minGQ 20 ‐‐maf 0.05 ‐‐max‐missing 0.8. Pairwise Weir & Cockerham's *F*_ST_ values (Weir and Cockerham 1984) were estimated in 50,000 bp windows across the genome using pixy v.1.2.11.beta1 (Korunes and Samuk 2021). Genome‐wide mean *F*_ST_ was calculated as the average across all windows.

For admixture analysis and principal component analysis (PCA), SNPs were filtered to remove low‐quality loci and physically linked SNPs using vcftools v0.1.16 (Danecek et al. 2011) with the following parameters: ‐‐max‐meanDP 87.5, ‐‐minDP 8, ‐‐minQ 20, ‐‐minGQ 20, ‐‐min‐alleles 2, ‐‐max‐alleles 2, ‐‐mac 1, ‐‐max‐missing 0.8, ‐‐maf 0.05, and ‐‐thin 1000. Individuals with high missing rates and an outgroup individual were removed as described above, resulting in a dataset of 223 individuals and 2097 SNPs. ADMIXTURE v1.3.0 (Alexander et al. 2009) was run with *K* values ranging from 1 to 10, and the model with the lowest cross‐validation error was selected. PCA was performed on the same dataset using the snpR v1.2.4 package (Hemstrom and Jones 2023) in R v4.4.1 (R Core Team 2024).

### 2.3 Morphological Measurements and *Ectodysplasin‐A* (*Eda*) Genotyping

To investigate morphological variation in Galician stickleback populations, we analyzed specimens from seven populations whose whole bodies were preserved in ethanol: Tines, Umia (downstream and upstream), Miñor (downstream and upstream), and Miño (downstream and upstream), all of which were collected in November 2016 (Table 1). We measured the following 11 morphological traits: standard length, head length, body depth, jaw length, snout length, gape width, eye diameter, first dorsal spine length, second dorsal spine length, left pelvic spine length, and pelvic girdle length, as described previously (Kitano et al. 2007), using a digital vernier caliper (Mitutoyo, Kanagawa, Japan) with a resolution of 0.01 mm (Table S2). Because morphological traits can exhibit sexual dimorphism (Kitano et al. 2007), sex was determined by PCR amplification of the *Idh* gene (Peichel et al. 2004), as described previously (Yamasaki et al. 2026). All morphological measurements were log‐transformed before PCA. PCA was performed in R v4.5.3 using the prcomp function. PC scores were analyzed using linear models including sex and lineage, with population nested within lineage.

Because all fish were smaller than 32 mm in standard length, below which plate development is incomplete (Bell et al. 2004), we did not count plate number; however, all individuals appeared to be of the low‐plated morph. Instead, we genotyped the major plate locus, *Ectodysplasin‐A* (*Eda*) (Colosimo et al. 2005). Genotyping at *Eda* was performed using the *Stn382* marker (Kitano et al. 2008; Yamasaki et al. 2026), which yields a 150‐bp fragment for the low‐plated allele and a 218‐bp fragment for the completely plated allele. PCR was conducted using KAPA2G Fast Multiplex (Roche, Wilmington, MA, USA), followed by electrophoresis on a 2% agarose gel.

### 2.4 Phylogenetic Comparison of Galician Sticklebacks With Global Populations

To place the two Galician lineages identified in this study within the global phylogeny of the three‐spined stickleback, we conducted additional WGS. Because the restriction enzyme used in a previous global phylogenetic study (Fang et al. 2018) was PstI and differed from those used here (EcoRI and BglII), the resulting RAD loci are not directly comparable, precluding the merging of the two datasets. To overcome this limitation, we performed WGS on a subset of Galician individuals (*n* = 27; Table S1) to obtain genome‐wide SNP data that could be integrated with previously published data (Fang et al. 2018). For WGS, libraries were prepared using the NEBNext Ultra II DNA Library Prep Kit for Illumina (New England Biolabs, USA) and sequenced on Illumina HiSeq X or NovaSeq X platforms in 150 bp paired‐end mode.

For global comparison, we used publicly available RAD‐seq data from 69 individuals (Fang et al. 2018) (Table S3) and WGS data from two Japanese *G. aculeatus* individuals, two *Gasterosteus nipponicus*, and one *Gasterosteus wheatlandi* (Yoshida et al. 2014) (Table S3). Sequence quality was assessed using FastQC v0.12.1 (Andrews 2010), and low‐quality reads were trimmed using Trimmomatic v0.39 (Bolger et al. 2014) with the following parameters: LEADING:3, TRAILING:3, SLIDINGWINDOW:4:15, and MINLEN:36. Filtered reads were mapped to the *G. aculeatus* version 5 reference genome (GCF_016920845.1) (Nath et al. 2021) using the BWA‐MEM algorithm implemented in BWA v0.7.17 (Li and Durbin 2009) with the ‐M option enabled.

Variant calling was conducted separately for the RAD‐seq and WGS datasets using mpileup in bcftools v1.19 (Danecek et al. 2021) with the minimum base quality filter applied using ‐Q 20 option. SNPs on chromosomes 9 and 19 were excluded because these chromosomes correspond to the sex chromosomes (Kitano et al. 2009), for which SNP calling is less reliable. For the RAD‐seq dataset, SNPs with a Phred‐scaled quality score (QUAL) ≧ 20, a mean site depth ≧ 3, a genotype depth ≧ 3, and a missing data proportion ≦ 0.2 were retained using vcftools v0.1.16 (Danecek et al. 2011) with the following option: ‐‐minQ 20 ‐‐min‐meanDP 3 ‐‐minDP 3 ‐‐max‐missing 0.8. Two individuals with missing data rates ≧ 0.2 (sample IDs: JPO and BUT) were removed. For the WGS dataset, SNPs with QUAL ≧ 20, mean site depth ≧ 10, genotype depth ≧ 10, and missing data proportion ≦ 0.2 were retained using vcftools v0.1.16 with the following options: ‐‐minQ 20 ‐‐min‐meanDP 10 ‐‐minDP 10 ‐‐max‐missing 0.8. After filtering, the RAD‐seq and WGS variant datasets were merged using the merge command in bcftools v1.19. After SNP filtration, 1,323,218 sites from 99 individuals were retained for genetic analysis.

To investigate which global *G. aculeatus* lineages the Galician populations belong to, we conducted PCA, ADMIXTURE, and phylogenetic analyses. PCA was performed using PLINK v1.9.0 (Purcell et al. 2007), and model‐based clustering was conducted using ADMIXTURE v1.3.0 (Alexander et al. 2009). Prior to analysis, SNPs in high linkage disequilibrium (*r* ^2^ > 0.5 within a 50‐SNP window) and those with minor allele counts ≦ 2 were removed using the following options: ‐indep‐pairwise 50 5 0.5 ‐mac 2, resulting in 843,446 sites from 96 individuals.

A phylogenetic tree was constructed using RAxML‐NG v0.9.0 (Kozlov et al. 2019). Only biallelic sites with a missing rate ≦ 0.01 were retained using vcftools with the following options: ‐‐min‐alleles 2 ‐‐max‐alleles 2 ‐‐max‐missing 0.99, resulting in 4986 sites from 99 individuals. The optimal nucleotide substitution model was determined using ModelTest‐NG v0.1.7 (Darriba et al. 2020), which identified the transversion model with a discrete Gamma model (TVM + G4) as the best‐fitting model based on the Akaike Information Criterion and Bayesian Information Criterion. Phylogenetic analysis was then conducted using RAxML‐NG with the following options: ‐model TVM + G4 + ASC_LEWIS ‐all ‐bs‐trees 1000 ‐bs‐metric fbp. *G. wheatlandi* was used as the outgroup.

## 3 Results

### 3.1 Two Lineages in Galician Populations

**FIGURE 2:**
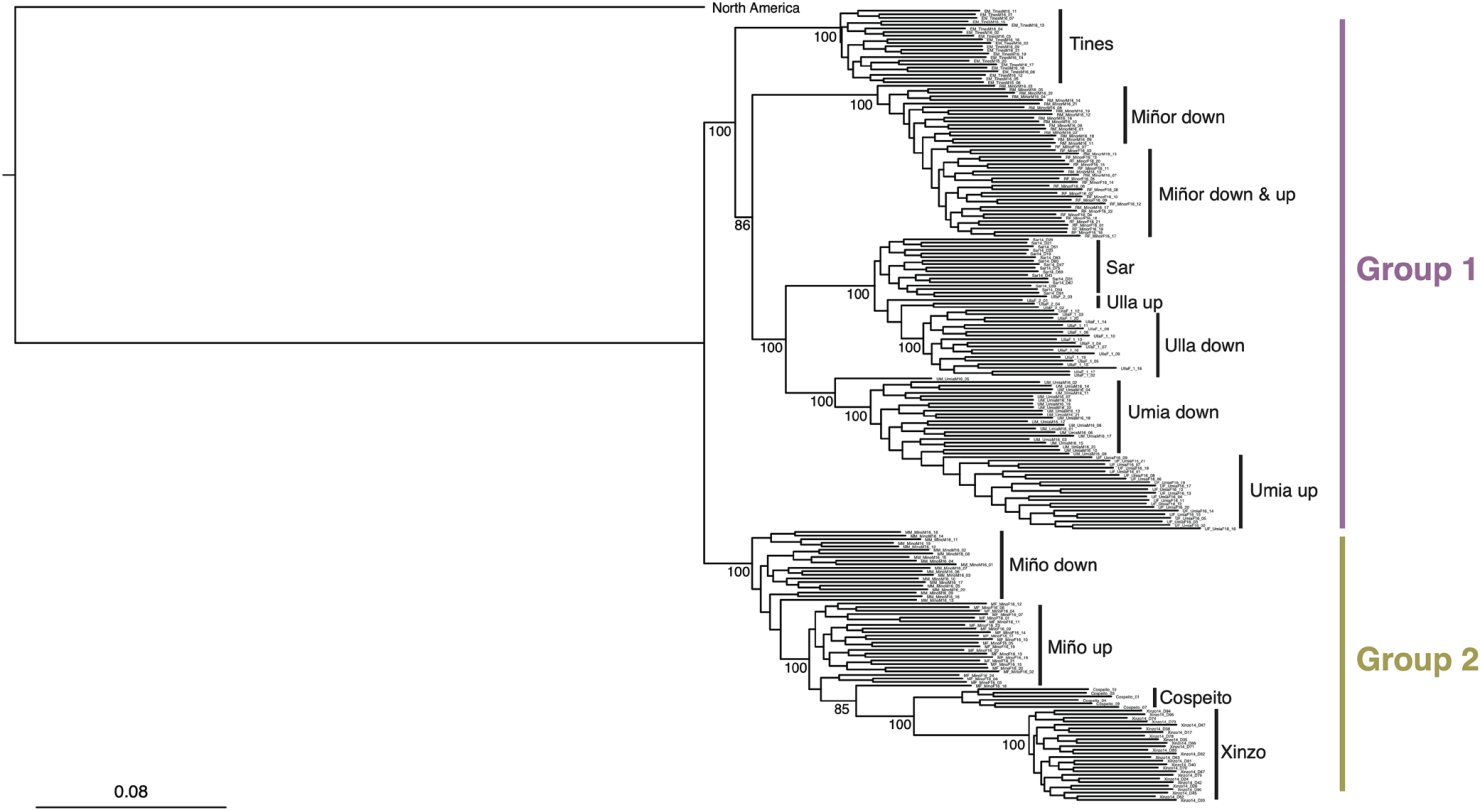
Phylogenetic tree of Galician stickleback populations. Bootstrap support values greater than 70 are shown at the nodes. The scale bar indicates the number of substitutions per site. Galician populations are divided into two lineages (Groups 1 and 2).

Phylogenetic analysis based on RAD‐seq data revealed two distinct lineages within the Galician populations, hereafter referred to as Groups 1 and 2 (Figure 2). Group 1 comprised populations from northern coastal regions, whereas Group 2 included populations spanning the full course of the Miño River (from Cospeito, near its source, to Miño down its estuary) as well as an inland site in the Limia River basin (Xinzo) (Figure 1A). The average *F*_ST_ between Groups 1 and 2 was 0.435, ranging from 0.224 to 0.605 (Figure S1).

Within river basins, populations clustered together, with low levels of genetic differentiation between upstream and downstream sampling sites (Figure 2). The average *F*_ST_ between upstream and downstream populations within rivers was 0.049, ranging from 0.032 to 0.066 (Figure S1).

**FIGURE 3:**
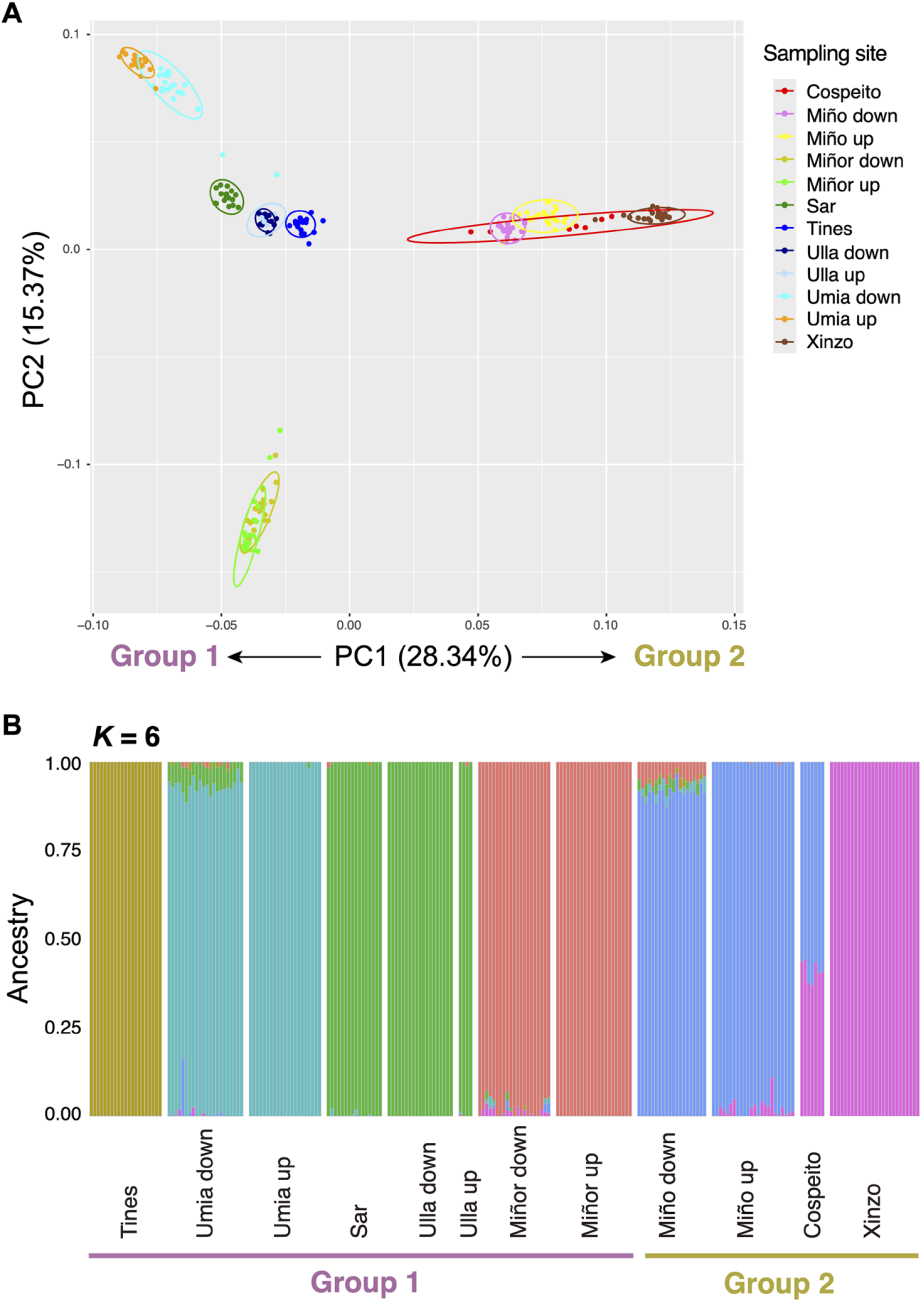
Genetic structure of Galician stickleback populations. (A) Principal component analysis (PCA) of genomic data. PC1 (28.34% of the variance) separates populations into two lineages (Groups 1 and 2). PC2 (15.37%) distinguishes the southernmost coastal populations (Miñor upstream and downstream) from the others. (B) ADMIXTURE results at *K* = 5, which.

PCA of genomic data also showed that Groups 1 and 2 populations were separated along PC1, which explains 28.34% of the total variance (Figure 3A). PC2, which explains 15.37% of the variance, further separates Group 1 populations, distinguishing the most southern coastal populations (Miñor upstream and downstream) from the others. ADMIXTURE analysis showed that individuals were mainly assigned to distinct genetic clusters corresponding to river systems (Figure 3B), with small traces of admixture between clusters in estuarine populations.

### 3.2 Morphological Variation and Abundant Low‐Plated *Eda* Allele

PCA of log‐transformed morphological measurements showed that PC1 explained 75.87% of the total variance and had uniformly high loadings (all negative) across all traits (Table S4), indicating that this axis represents overall body size. Lower PC1 values correspond to larger individuals. In contrast, PC2 explained 6.45% of the variance and captured shape variation, with positive loadings for eye diameter and negative loadings for dorsal and pelvic spine lengths (Table S4).

**FIGURE 4:**
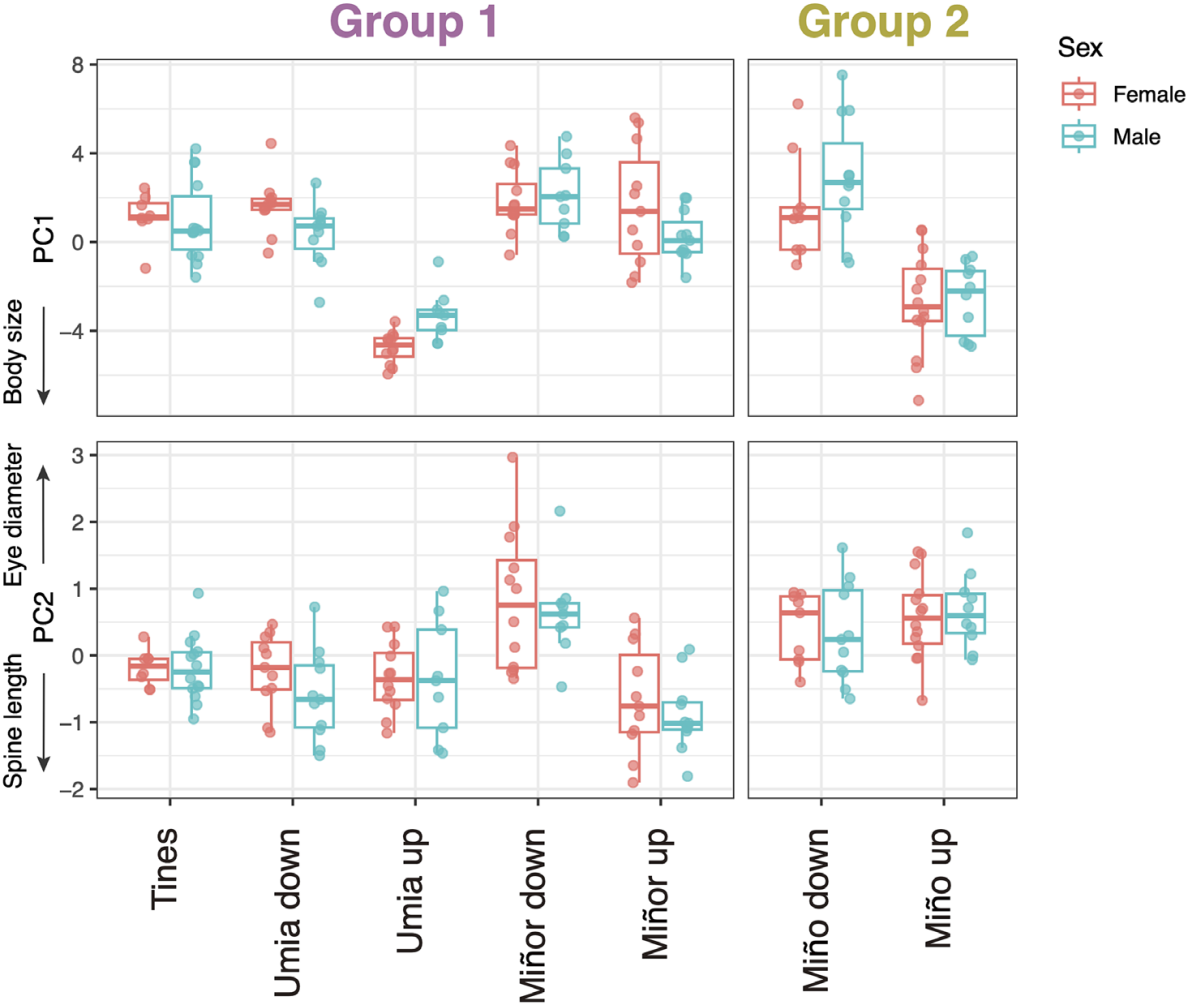
Morphological variation in Galician stickleback populations. Principal component 1 (PC1) and PC2 scores for individuals grouped by sampling location and lineage. PC1 and PC2 explain 75.87% and 6.45% of the total variance, respectively. Males and females are shown separately. Boxplots indicate medians and interquartile ranges, and points represent individual samples.

For PC1, ANOVA showed no significant effect of sex (*F*_1,144_ = 1.78, *p* = 0.185) and a marginal effect of lineage (*F*_1,144_ = 3.23, *p* = 0.074). Visual inspection of the data suggested that differences between lineages were modest and rather varied among sampling locations (Figure 4A).

For PC2, ANOVA revealed a significant effect of lineage (*F*_1,144_ = 35.48, *p* = 1.89 × 10^−8^) (Figure 4B), with Group 2 showing higher PC2 values than Group 1. This pattern is consistent with Group 2 individuals having relatively larger eyes and shorter spines, although the limited number of populations included in the morphological analyses prevents a clear separation of lineage and population effects. In contrast, the effect of sex was not significant (*F*_1,144_ = 2.35, *p* = 0.128). The effect of sampling location nested within lineage was also significant (*F*_5,144_ = 12.55, *p* = 4.06 × 10^−10^), indicating additional variation among populations within each lineage.

Genotyping at the *Eda* locus showed that all individuals carried the low‐plated allele, except for a single individual from Tines, which was heterozygous.

### 3.3 Galician Populations Belong to the Southern European Clade

**FIGURE 5:**
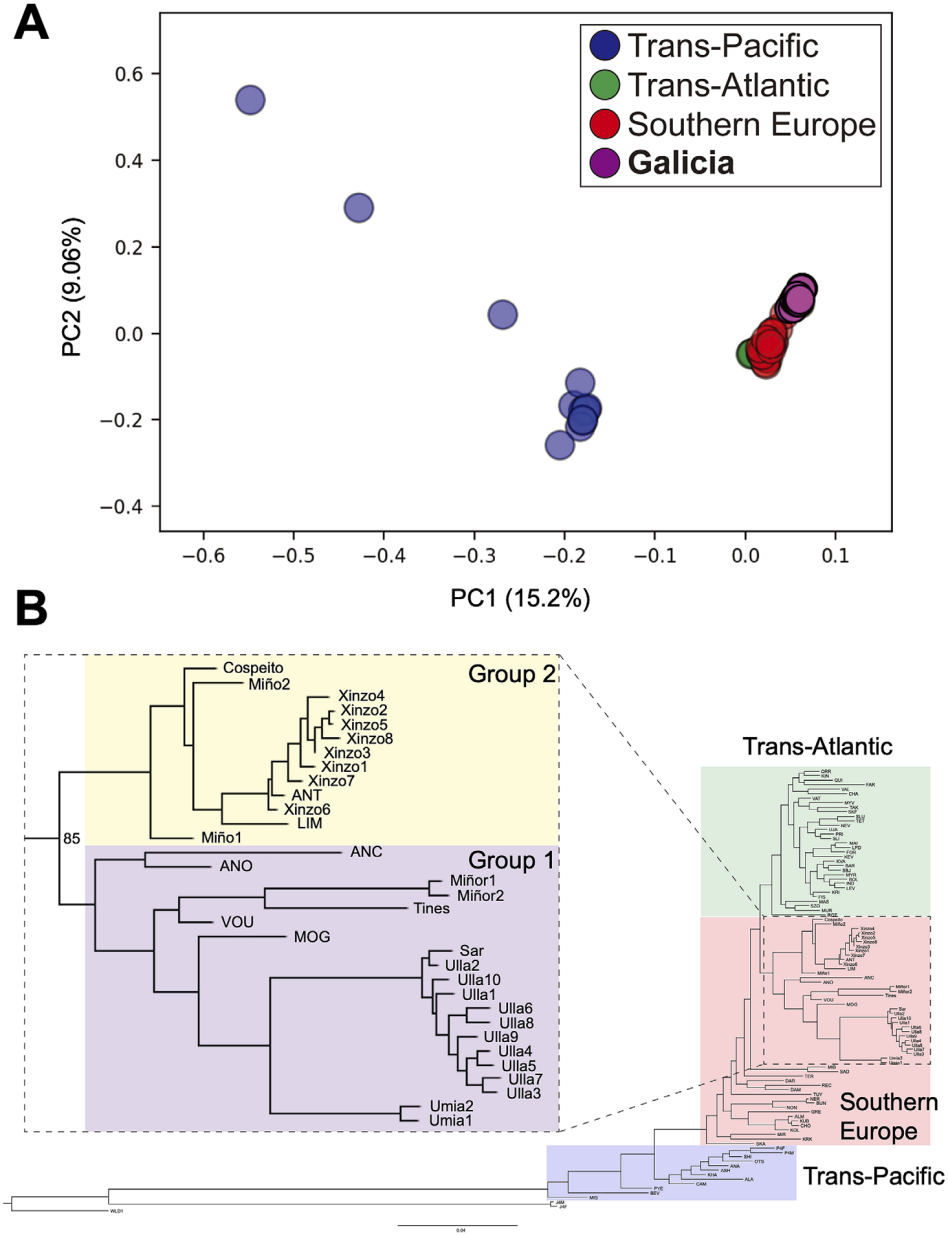
Galician sticklebacks belong to the Southern European Clade. (A) Principal component analysis (PCA) of genomic data including Galician and global stickleback populations. PC1 and PC2 explain 15.2% and 9.06% of the total variance, respectively. (B) Phylogenetic tree including Galician and global stickleback populations.

PCA of genomic data including global populations showed that all Galician sticklebacks were genetically closer to the Southern European Clade than to the Trans‐Atlantic and Trans‐Pacific Clades (Figure 5A). Phylogenetic analysis also placed all Galician individuals within the Southern European Clade (Figure 5B). Among Portuguese coastal populations, all were assigned to Group 1 except for the northernmost population near the Spanish border (LIM), which is in the same river basin as the Xinzo population and clustered with Group 2. Interestingly, a population from eastern Spain near Valencia was also included in Group 2 (Figure 5B). This clustering pattern was further supported by ADMIXTURE analysis, with the lowest cross‐validation error (0.25840) at *K* = 5 (Figure S2).

## 4 Discussion

Our phylogenomic analysis revealed the presence of two genetically distinct lineages within Galicia, highlighting the importance of sampling underrepresented geographic regions in studies of stickleback evolution. Because samples belonging to the same lineage were collected in different years, the major phylogeographic patterns observed here are unlikely to be explained solely by temporal variation in sampling. The origin of these two lineages remains unclear, but several non‐mutually exclusive explanations can be considered. One possibility is that they represent remnants of multiple glacial refugia within the Iberian Peninsula. Under the “refugia within refugia” hypothesis, climatically stable regions maintained isolated populations during Pleistocene climatic oscillations, leading to deep genetic divergence among lineages (Gómez and Lunt 2007). Consistent with this possibility, mitochondrial DNA analyses of Iberian three‐spined sticklebacks identified divergent Portuguese and Mediterranean lineages and suggested that these lineages diverged before the last glaciation, indicating the presence of multiple deeply divergent genetic lineages within the Iberian Peninsula (Sanz et al. 2015). The substantial differentiation observed between Groups 1 and 2 (mean *F*_ST_ = 0.435) is consistent with long‐term historical isolation. Whether these lineages have remained isolated throughout their history or have experienced periods of secondary contact remains unknown. A second possibility is that differences among populations in their degree of connectivity with marine environments have contributed to the observed structure. Further analyses of demographic history and gene flow will be necessary to clarify the processes underlying this divergence.

Although morphological differences were detected between the two lineages, the limited number of populations analyzed prevents us from disentangling lineage‐level effects from population‐level variation. Consequently, the observed differences should be interpreted cautiously and do not necessarily indicate consistent morphological divergence between the two lineages. Future studies incorporating additional populations across a broader geographic range will be important for evaluating whether lineage identity consistently influences patterns of phenotypic variation. A population from the Ulla River has been widely used in behavioral and evolutionary studies, which have shown that differing patterns of sexual and natural selection, compared with northern populations, have shaped morphological and behavioral phenotypes in this population (Kim and Velando 2014, 2015, 2016, 2020). Our results indicate that this population belongs to Group 1 and therefore represents a subset of the genetic diversity present in this region. Comparative studies across both lineages may therefore provide valuable insights into how genetic structure shapes phenotypic variation.

Both Galician lineages belong to the Southern European Clade of the three‐spined stickleback. Previous studies have shown that southern European freshwater populations share alleles that are commonly found in other global freshwater populations, suggesting that standing genetic variation contributes to repeated evolution of freshwater‐associated phenotypes (Coll‐Costa et al. 2024). Consistent with this, we found that most Galician individuals carry the low‐plated *Eda* allele that has been associated with freshwater adaptation in many regions of the species' range (Colosimo et al. 2005). This is consistent with a previous study (Fernández et al. 2000), which reported that Galician sticklebacks from the Miño and Limia rivers are predominantly low‐plated, with only a few individuals bearing keels in inland populations. The near fixation of the low‐plated *Eda* allele is notable because at least some populations occur in habitats that retain some degree of connectivity to marine environments. The processes responsible for this pattern remain unclear, but one possibility is that gene flow from marine populations is limited in this region. Further ecological and demographic data will be required to evaluate this hypothesis.

Our WGS data provide a valuable resource for future studies investigating whether other freshwater‐adaptive alleles identified in different regions (Fang et al. 2020; Jones, Grabherr, et al. 2012) are also present in Galician populations and whether their frequencies differ between the two lineages. An important limitation of the present study is the lack of detailed ecological information for most sampling sites. In particular, salinity regimes, connectivity with marine environments, and migratory behavior remain largely unknown. Consequently, the ecological factors underlying the observed genetic and morphological variation cannot currently be evaluated. Future studies integrating genomic data with ecological characterization of habitats will be necessary to understand the relationship between environmental variation and lineage divergence in Galician sticklebacks.

In conclusion, our study has revealed previously unrecognized fine‐scale phylogeographic structure within Galicia and places these populations within the Southern European Clade of sticklebacks. These findings demonstrate that even well‐studied model systems can harbor substantial hidden genetic structure in under‐sampled regions. Galician populations therefore provide an important system for investigating how geographic structure shapes patterns of morphological and behavioral differentiation.

## Author Contributions

**Yo Y. Yamasaki:** conceptualization (equal), data curation (equal), formal analysis (equal), funding acquisition (equal), investigation (equal), visualization (equal), writing – original draft (equal). **Yu Endo:** formal analysis (equal), investigation (equal), visualization (equal), writing – original draft (equal). **Naomi Musto:** formal analysis (equal), investigation (equal), writing – review and editing (supporting). **Juan Galindo:** conceptualization (equal), investigation (equal), resources (equal), writing – review and editing (supporting). **Alberto Velando:** conceptualization (equal), investigation (equal), resources (equal), writing – review and editing (supporting). **Sin‐Yeon Kim:** conceptualization (equal), investigation (equal), resources (equal), writing – review and editing (supporting). **Atsushi J. Nagano:** investigation (equal), writing – review and editing (supporting). **Asano Ishikawa:** conceptualization (equal), funding acquisition (equal), investigation (equal), writing – review and editing (supporting). **Jun Kitano:** conceptualization (equal), data curation (supporting), funding acquisition (equal), investigation (equal), project administration (lead), supervision (lead), visualization (supporting), writing – original draft (equal), writing – review and editing (lead).

## Funding

This research was supported by Japan Society for the Promotion of Science (JSPS) KAKENHI: 22KK0105 and 20J01503 to Y.Y.Y., 19KK0187 to A.I., and 25K24522 to J.K. Y.E. was a JSPS postdoctoral fellow. This was also supported by Xunta de Galicia (ED431C 2024/22), Centro singular de investigación de Galicia accreditation 2024–2027 (ED431G 2023/07) and “ERDF A way of making Europe”.

## Conflicts of Interest

The authors declare no conflicts of interest.

## Supporting information


**Figure S1:** Pairwise FST between populations.
**Figure S2:** Results of ADMIXTURE at *K* = 5, which showed the lowest cross‐validation error.

**Table S1:** Sequence accession numbers.
**Table S2:** Morphological data shown in mm.
**Table S3:** RAD and WGS data of global populations taken from previous studies.
**Table S4:** Principal component loadings for morphological traits.

## Data Availability

Sequence data have been uploaded to DDBJ (PRJDB42300 for RAD‐seq data and PRJDB42308 for WGS data). Morphological data are available in the Tables S1–S4.
