# Supplementary material for: Two Genetically Distinct Lineages in Galician Populations of the Three‐Spined Stickleback (Gasterosteus aculeatus)
Source: Ecol Evol. 2026 Jul 19;16(7):e74051. doi: 10.1002/ece3.74051 (PMC13381065; doi:10.1002/ece3.74051)
Supplement: Supplementary file 1 — Figure S1: Pairwise FST between populations. Figure S2: Results of ADMIXTURE at K = 5, which showed the lowest cross‐validation error. [file ECE3-16-e74051-s001.pdf]

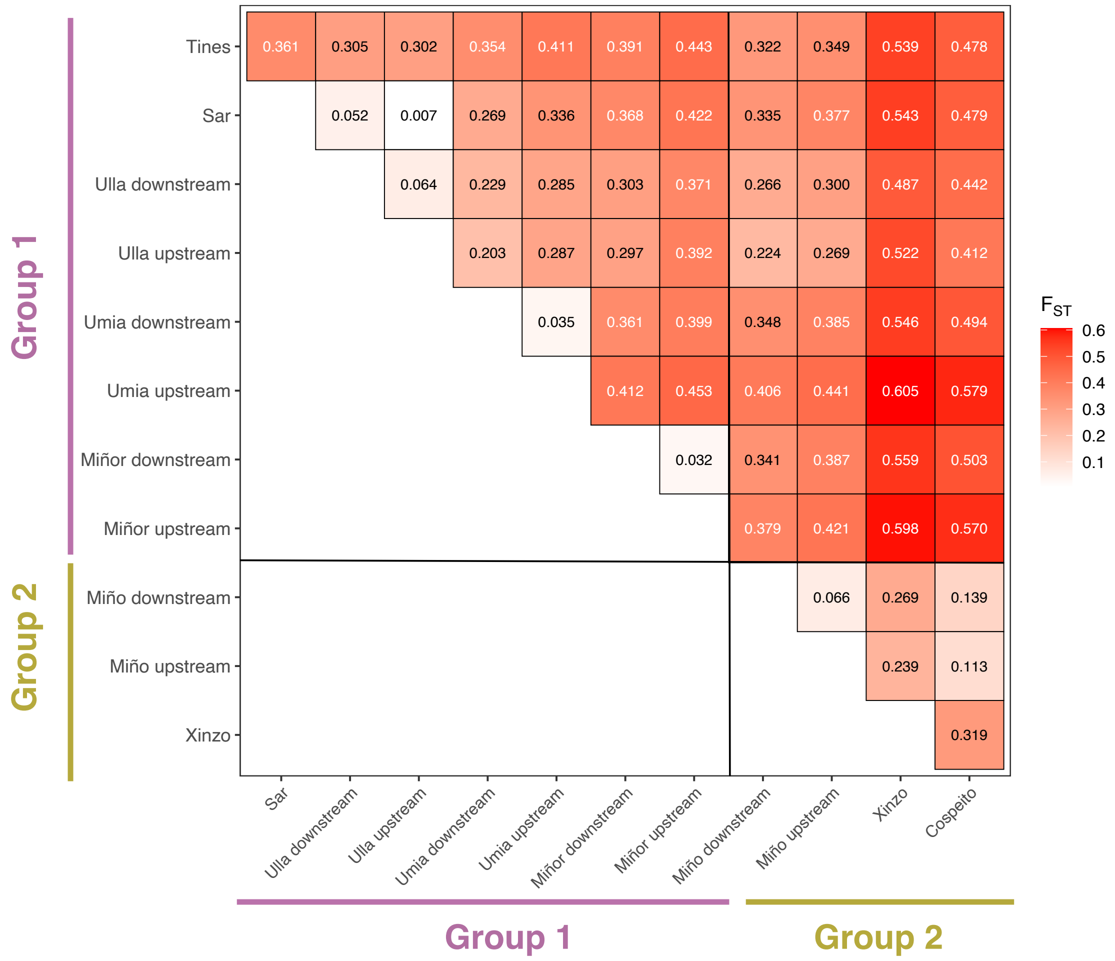

Figure S1 | Pairwise F_{ST} between populations.

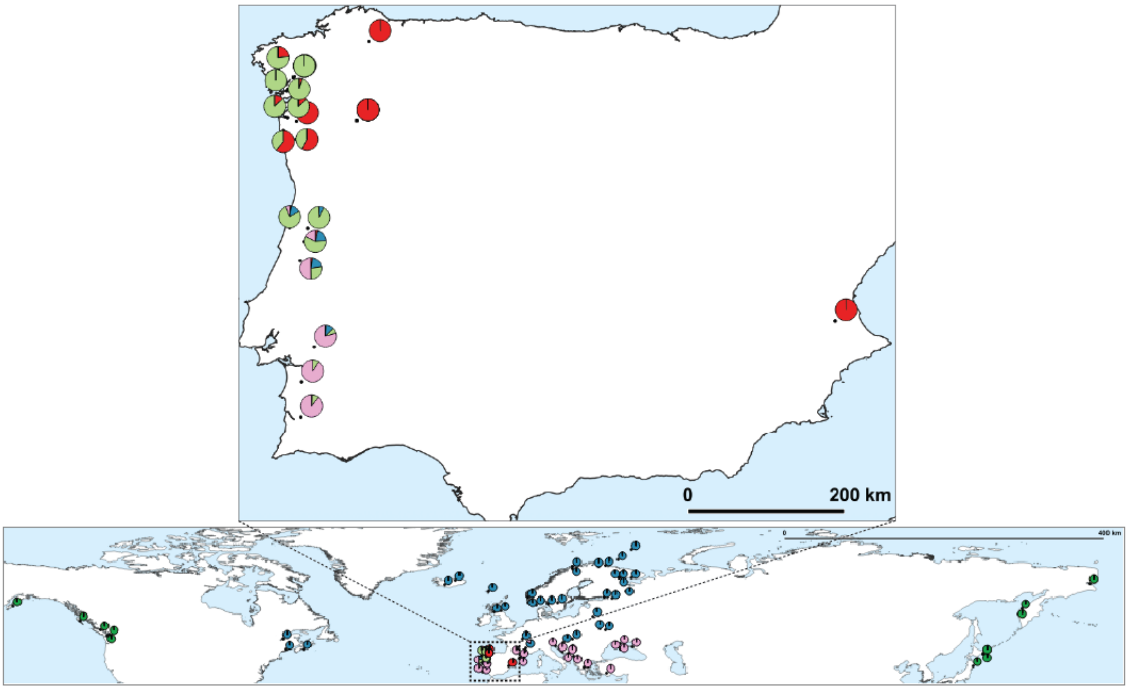

Figure S2 | Results of ADMIXTURE at *K* = 5, which showed the lowest cross-validation error.
